# Evolution under strong balancing selection: how many codons determine specificity at the female self-incompatibility gene *SRK *in Brassicaceae?

**DOI:** 10.1186/1471-2148-7-132

**Published:** 2007-08-06

**Authors:** Vincent Castric, Xavier Vekemans

**Affiliations:** 1 Laboratoire de génétique et évolution des populations végétales UMR CNRS 8016, Cité Scientifique, Université des Sciences et Technologies de Lille 1, 59655 Villeneuve d'Ascq cedex, France

## Abstract

**Background:**

Molecular lock-and-key systems are common among reproductive proteins, yet their evolution remains a major puzzle in evolutionary biology. In the Brassicaceae, the genes encoding self-incompatibility have been identified, but technical challenges currently prevent detailed analyses of the molecular interaction between the male and female components. In the present study, we investigate sequence polymorphism in the female specificity determinant *SRK *of *Arabidopsis halleri *from throughout Europe. Using a comparative approach based on published *SRK *sequences in *A. lyrata *and Brassica, we track the signature of frequency-dependent selection acting on these genes at the codon level. Using simulations, we evaluate power and accuracy of our approach and estimate the proportion of codon sites involved in the molecular interaction.

**Results**

We identified several members of the S-gene family, together with 22 putative S-haplotypes. Linkage to the S-locus and the presence of a kinase domain were formally demonstrated for four and six of these haplotypes, respectively, and sequence polymorphism was extremely high. Twenty-five codons showed signs of positive selection in at least one species, and clustered significantly (but not exclusively) within hypervariable regions. We checked that this clustering was not an artifact due to variation in evolution rate at synonymous sites. Simulations revealed that the analysis was highly accurate, thus providing a reliable set of candidates for future functional analyses, but with an overall power not higher than 60 %. Assuming similar power, we infer from our results that about 23% of all codons in the S-domain may actually be involved in recognition. Interestingly, while simulations demonstrated that this comparison remained reliable even at very high levels of divergence, codons identified in Brassica had higher posterior rates of non-synonymous to synonymous substitutions than codons identified in *A. halleri or A. lyrata*, possibly suggesting more intense selection in Brassica.

**Conclusion**

The signature of balancing selection can be identified reliably at the codon level even in cases of very high sequence divergence, provided that a sufficiently large set of sequences are analyzed. Altogether, our results indicate that a large proportion of codons may be involved in recognition and confirm the particular importance of hypervariable regions. The more intense signature of positive selection detected in Brassica suggests that allelic diversification in this genus was very recent, possibly following a recent demographic bottleneck.

## Background

Molecular lock-and-key systems are common among reproductive proteins and receptor-hormone complexes [1]. Because the lock and the key must match in such systems, any evolutionary novelty at one component must be accompanied by a matching novelty at the other. This coevolution constraint has made the generation of new combinations a major -and as yet unsolved- puzzle in evolutionary biology [2-4]. Understanding molecular details of the interaction can give important insight into the evolutionary dynamics of these systems [5]. In particular, the proportion of sites involved in recognition and potentially able to alter the interaction is crucial.

Self-incompatibility (SI) systems are fascinating molecular lock-and-key mechanisms preventing selfing in species from over half of all angiosperm families [6]. In the Brassicaceae, the lock and the key are encoded by *SRK *and *SCR *respectively, two highly polymorphic and closely linked genes. *SCR *encodes a small protein expressed in anthers and deposited in the pollen exine layer, while *SRK *encodes a transmembrane serine/threonine kinase with allele-specific affinity for *SCR *proteins.

*SRK *is an integral component of the plasma membrane of the stigma epidermis and is displayed with its glycosylated N-terminal S-domain external to the cell. When activated by binding to its cognate *SCR *protein [7,8], it prevents selfing by triggering a downstream regulatory pathway that ultimately inhibits pollen tube germination.

Although the recognition between *SCR *and *SRK *has been demonstrated at the molecular level, the regions of these molecules important for specificity recognition have remained elusive. In *SCR*, a domain swapping experiment in Brassica between allele *SCR*_6 _and allele SCR_13 _resulted in a switch from SCR_6 _to SCR_13 _specificity, but failed to switch specificity when the reverse swapping (SCR_13 _domains to a SCR_6 _backbone) was performed [9]. These results suggest the intriguing possibility that functionally important sites may not be the same in the two alleles investigated.

Clearly, the analysis of more alleles will now be required to confirm the role of these domains (see also [10-12]). A second experimental approach in *SCR *relied on the functional comparison between pairs of closely related alleles in *Brassica rapa *and *B. oleracea*. [13] demonstrated that pollen specificity was partly and completely altered between *SCR *copies differing by four and eight amino-acid differences respectively, thus providing strong candidates for functionally important codons. In *SRK*, technical challenges associated with crystallizing trans-membrane proteins have hampered the resolution of the three-dimensional structure of the protein, such that sites involved in binding *SCR *(and thus determining specificity) are currently unknown. Hence, specificity-determining nucleotides along the *SRK *sequence have mainly been inferred from the observation that stretches of nucleotides have unusually elevated replacement site polymorphism as compared to the rest of the S-domain. Such hyper-variable (HV) regions have been observed in Brassica [14-16], *Raphanus sativus *[17] and *Arabidopsis lyrata *[18]. [19] and [20] demonstrated that these regions are particularly conserved among functionally equivalent allelic copies of *BoSRK02 *and *BoSRK13 *in *B. oleracea*, and [21] further showed that pairs of closely related alleles in *B. rapa *and *B. oleraceae *with identical recognition specificity typically exhibit very few differences in HV regions. More recently, [22] provided further support for a functional role of these regions by reporting that alleles in *A. halleri*, *A. lyrata *and *A. thaliana *that were probably inherited from a common ancestor have fewer amino acid differences in these regions than in the rest of the gene.

A limit to this "direct comparison" approach to pinpoint functionally important sites is that it typically relies on a low number of naturally occurring variants and does not integrate over evolutionary times. An alternative approach is based on the assumption that sites involved in the interaction evolve under specific selective pressures. In particular, while purifying selection at a codon site is expected to decrease the rate of non-synonymous to synonymous evolution (*d*_N_/*d*_S _ratio, hereafter *ω*), positive and balancing selections are expected to increase this ratio. Tracking the signature of natural selection at the codon level can thus reveal functionally important sites along a sequence (see *e.g*. [23]). As initially demonstrated by Wright [24], loci governing SI are expected to experience negative frequency-dependent selection, a form of strong balancing selection whereby low frequency *SCR*-*SRK *combinations enjoy a selective advantage over alleles present at higher frequency because they encounter their cognate allele only rarely. Consequently, substitutions affecting allelic specificity are expected to enter a population more often than substitutions not affecting specificity, leading to a higher rate of non-synonymous than synonymous substitution at codons functionally determining specificity. In line with this expectation, [16] found higher *d*_N_*/d*_S _in Brassica HV regions than in the rest of the protein (1.37 within *vs*. 0.41 outside HV regions), thus providing further support for their role in specificity.

Similarly, [25] showed that HV regions in Brassica are enriched in sites with elevated *d*_N_*/d*_S _ratios as compared to the rest of the protein.

A recent paper estimated in Brassica that 27 of the *c.a*. 423 codon sites (= 6.38%) of the S-domain of *SRK *(precise length varies) are likely to evolve under recurrent positive selection events [26]. However, the very long expected time to coalescence of alleles at *SRK *translates into very high levels of diversity, which can saturate the signal of divergence. This may bring uncertainty into the phylogeny of *SRK *alleles, causing potential bias and lowering the power of the detection method. As shown by [27], the power of the analysis indeed decreases with sequence divergence. The possibility thus exists that these codons represent a very small proportion of sites involved in specificity. Moreover, *SRK *diversity in Brassica is unusual among the Brassicaceae in that alleles at this locus cluster into two very distinct and highly divergent allelic classes. Brassica thus only shows a subset of the total molecular diversity found at this gene in the related *A. lyrata *[18], and we recently reported at least two deeply divergent *SRK *alleles in the closely related *A. halleri *[22]. There has currently been no evaluation of how the particular allelic topology in Brassica may affect the power and accuracy of the method. As reported in [27], increasing the number of lineages (*e.g*. by including widely divergent lineages from Arabidopsis species) is one of the most efficient ways to increase the statistical power of the method.

In the present paper, we aim at estimating the number of codons determining allelic specificity at the pistil gene involved in self-incompatibility in the Brassicaceae, and at testing whether these codons cluster specifically within the well-known hypervariable regions of the S-domain, through an analysis of a large data set of sequences belonging to three taxa. We report nucleotide sequences for 22 new *SRK *alleles in *A. halleri*, including about half of the S-domain for all alleles and the kinase domain for six alleles. We provide evidence for some of these sequences that they do indeed belong to *SRK *and compare sequence polymorphism between *A. halleri*, *A. lyrata *and Brassica. We use a maximum likelihood method to identify codons along the gene that evolved under positive selection and compare the number, location and rate of evolution of such sites in the three taxa. Through simulations we demonstrate that the analysis is highly accurate, and estimate that the power approaches 60%. Since 25 codons exhibit signs of positive selection in at least one species, we estimate that almost a quarter of the sites in the sequence (23%) could be involved in recognition. Our analysis also provides additional support for a functional role of hypervariable regions along the gene, and suggests that the high diversity observed in the kinase domain is due to tight linkage to the S-domain rather than to direct exposure to positive selection.

## Results

### Sequences obtained

**Figure 1:**
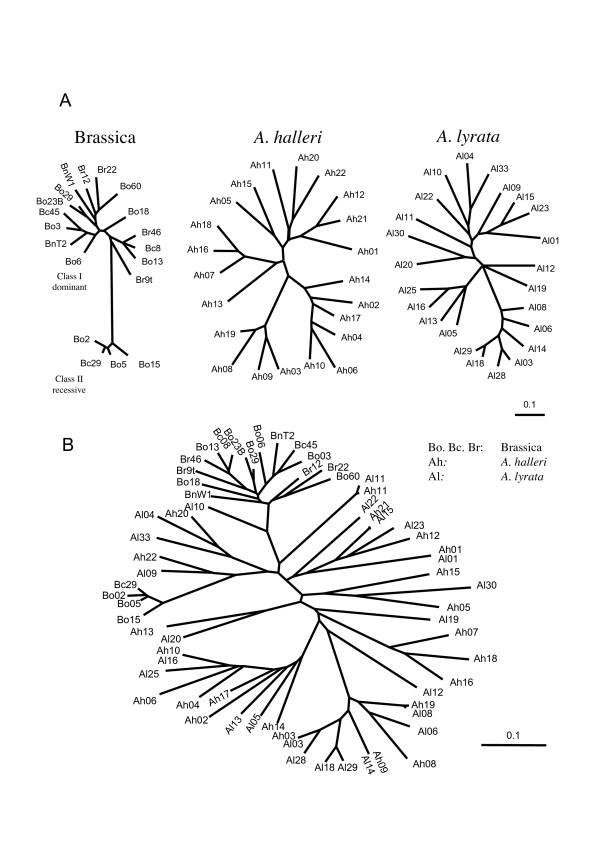
**Phylogeny of *SRK *alleles in *A. halleri*, *A. lyrata *and Brassica**. A: 50% majority rule consensus phylogeny obtained independently for each taxon using MrBayes. B: Combined maximum likelihood phylogeny, used to simulate sequence evolution in the Evolver program. Note the scale difference between the single-species and the combined trees. Allele names have been abbreviated for clarity.

According to phylogenetic clustering with published sequences in *A. lyrata*, the sequences obtained corresponded to twenty new putative *A. halleri *S-alleles and to six separate loci belonging to the Brassicaceae S gene family, namely *Aly7*, *Aly8*, *Aly9*, *Aly10.1*, *Aly13-2 *and *Aly13-7 *(Fig. 1A and Table 1; [28]). Linkage to a kinase domain was demonstrated for alleles *AhSRK03*, *04, 06, 07, 10 *and *18 *(Table 2). For 12 of the 22 putative S-alleles in *A. halleri*, we found a matching sequence with high similarity at the S-locus in *A. lyrata *(Table 2). Sequences from this study have GenBank accession numbers EU075124–EU075143, see additional file 1.

**Table 1:** Sampling locations of individuals analyzed in this study and inventory of the sequences identified from the S-locus and from separate loci belonging to the Brassicaceae S gene family.

Population	latitude	Longitude	Number of plants analyzed	S-locus sequences	Other S gene family sequences
France					
Auby	03°03'	50°25'	17	*AhSRK01*, *02, 03, 04, 12*	*Aly13-2, Aly13-7, Aly7, Aly8, Aly9, Aly10.1*
Germany					
D01	12°09'88	49°10'64	1	*AhSRK05*, *06*	*Aly9*
D02	12°09'52	49°11'31	1	*AhSRK07*	*Aly13-2, Aly9*
D06	12°49'78	49°17'42	1	*AhSRK05*,*09*	*Aly8, aly9*
D08	10°29'04	51°53'79	2	*AhSRK01*, *05*, 11	*Aly9*
D09	10°25'16	51°53'46	1	*AhSRK03*, *10*	*Aly9*
D11	10°21'95	51°51'27	1	*AhSRK01*, *02*	
Belgium					
B01	06°40'	50°29'63	1	*AhSRK13*	*Aly9*
Poland					
PL02	18°56'67	50°29'68	1	*AhSRK03*, *14*	*Aly9*
PL04	18°55'79	50°29'98	1	*AhSRK15*	
PL06	19°01'52	50°16'95	1	*AhSRK14*, *16*	*Aly9*
Slovakia					
SK02	21°07'81	48°46'17	1	*AhSRK08*	
Czech republic					
CZ04	13°45'841	49°02'87	1	*AhSRK18*	
CZ05	13°46'39	48°59'25	2	*AhSRK18*, *19*	*Aly8, Aly9*
CZ08	13°48'	48°57'	1	*AhSRK05*	*Aly9*
CZ14	12°42'92	49°28'37	1	*AhSRK17*, 21	*Aly9*

Note : since different primer combinations amplifying different sets of alleles were used for different individuals, the data from this table cannot be used to compare allele frequencies among populations.

**Table 2:** Characteristics of the *A. halleri *alleles.

Allele	Demonstrated presence of a kinase domain (length of intron 1)	Existence of a closely related *A. lyrata *allele	Evidence for linkage to incompatibility phenotype (P)	Group (according to Prigoda, Nassuth, and Mable 2005)
*AhSRK01*		*AlSRK01*	Yes	A1
*AhSRK02*		*AlSRK17*		A3
*AhSRK03*	Yes (504 bp)	*AlSRK03*		B
*AhSRK04*	Yes (255 bp)	*AlSRK37*	Yes	A3
*AhSRK05*		*AlSRK34*		-
*AhSRK06*	Yes (588 bp)			A3
*AhSRK07*	Yes (678 bp)			-
*AhSRK08*				B
*AhSRK09*		*AlSRK14*		B
*AhSRK10*	Yes (411 bp)	*AlSRK16*		A3
*AhSRK11*		*AlSRK11*		A2
*AhSRK12*			Yes	A2
*AhSRK13*				-
*AhSRK14*				A3
*AhSRK15*			Yes	-
*AhSRK16*		*AlSRK31*		-
*AhSRK17*				A3
*AhSRK18*	Yes (788 bp)	*AlSRK39*		-
*AhSRK19*		*AlSRK08*		B
*AhSRK20*				A2
*AhSRK21*		*AlSRK15*	Yes	A2
*AhSRK22*				A2

### Co-segregation of four putative *SRK *alleles and linkage to incompatibility phenotype

Offspring from a controlled interspecific cross between an *A. lyrata *plant with genotype *AlSRK14/AlSRK21 *and an *A. halleri *pollen donor with genotype *AhSRK15/AhSRK21 *demonstrated strict co-segregation of the two pairs of putative *SRK *alleles, although with segregation ratios substantially different from the expected 1:1 ratio: *AlSRK14/AlSRK21 *with a ratio 39:14 and *AhSRK15/AhSRK21 *with a ratio 43:17. Segregation bias among S-alleles has already been reported in [29].

Incompatibility reactions were tested for ten progeny plants from the interspecific cross, including 2 or more of each of the four *SRK *genotypes. 108 pollinations involved plants whose putative genotypes predict compatibility (no shared *SRK *sequences), and for these fruit set was 90.7%. 104 pollinations involved plants whose putative genotypes predict cross-incompatibility, and the mean fruit set was 3.8%. These results provided direct evidence that *AhSRK15 *and *AhSRK21 *are functional *SRK *alleles in *A. halleri*, and confirmed that *AlSRK14 *and *AlSRK21 *are functional *SRK *alleles in *A. lyrata*.

### Sequence polymorphism

**Figure 2:**
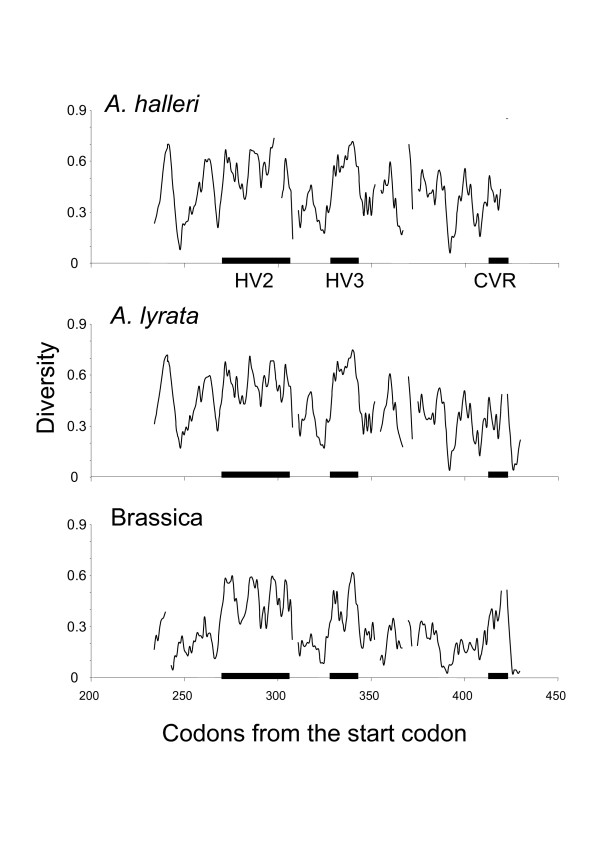
**Sliding window analyses of amino acid diversity for *A. halleri, A. lyrata *and Brassica**. The window was 5 amino acid wide and sled with a step of 1 amino acids. Horizontal black bars represent the three hypervariable regions HV2, HV3 and CVR.

The region sequenced spans three of the four different hypervariable regions (HV2, HV3 and CVR) defined in Brassica [30]. Aligned protein sequences were variable in length, differing by as much as 6 amino acid residues (180 residues for *AhSRK12 *and 186 for *AhSRK03, 07, 09, 16 *and *19*). There was no stop codon in any of the sequences. Only 29 of the 186 amino acid sites were conserved over all sequences, 28 being identical in state with the Brassica sequence *BoSRK60*. Twelve of these conserved residues were cystein residues at identical locations as those described in Brassica by [30]. The 22 sequences contained on average 3.36 predicted N-linked glycosylation sites per sequence. Of these, two sites (N246 and N391) were highly conserved, being shared by all 22 sequences with a single exception for the latter (absent in *AhSRK14*). A third site (N316) was partially conserved, being shared by 6 sequences. The sliding window analysis revealed that the three hypervariable regions defined in Brassica also showed elevated diversity in *A. halleri *and *A. lyrata *(Fig. 2). However, since the baseline nucleotide diversity outside hypervariable regions was also strikingly higher in *A. halleri *and *A. lyrata *than in Brassica, the peaks of diversity in hypervariable regions tended to be less pronounced.

Diversity also varied along the different exons of the gene. Non-synonymous diversity was higher in the S-domain (in *A. halleri, π*_*N *_= 0.310 in exon 1; Table 3) than in the rest of the gene, and tended to decrease with increasing physical distance from the S-domain, reaching as low a value as *π*_*N *_= 0.054 in exon 6. In contrast, although synonymous diversity was higher in the S-domain (*π*_*S *_= 0.819, Table 3) than in the kinase domain (average *π*_*S *_= 0.529), diversity remained high throughout the kinase domain (e.g. *π*_*S *_= 0.732 in exon 5), such that no decrease was apparent with increasing physical distance from the S-domain. We found no evidence in any of the three species for a significant correlation between linkage disequilibrium (LD) and distance between variable sites (*p *> 0.05), indicating no strong effect of recombination between *SRK *haplotypes.

**Table 3:** Diversity estimates for the coding sequence of *SRK *in *A. halleri*

	S-domain	Trans-membrane Domain	Kinase domain			
	exon 1	exon 2	exon 3	exon 4	exon 5	exon 6

Nb sequences	22	6	6	6	3	2
*S*	107.5	24.7	35.9	28.7	13.0	34.1
*N*	399.6	83.3	123.1	103.3	47.0	115.9
*π*	0.388	0.312	0.359	0.237	0.300	0.107
*π*_*S*_	0.819	0.432	0.437	0.625	0.732	0.325
*π*_*N*_	0.310	0.271	0.219	0.168	0.170	0.054

*S *and *N *are, respectively, the average numbers of potentially synonymous and non-synonymous sites analyzed. *π, π *_*S *_*and π *_*N *_are total, synonymous and non-synonymous diversities, respectively. All diversity estimates were Jukes & Cantor corrected.

### Sites under positive selection

**Figure 3:**
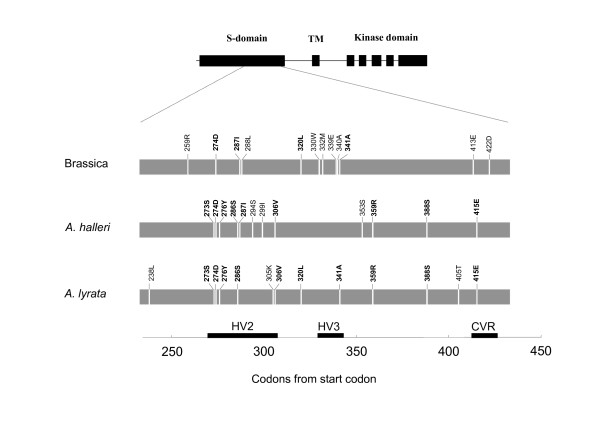
**Intron-exon structure of *SRK *and sites identified as evolving under positive selection in Brassica, *A. halleri *and *A. lyrata***. Sites 270–306, 328–343, and 413–423 correspond to hypervariable regions HV2, HV3, and CVR respectively. Codons identified by more than a single species are figured in bold.

In all three taxa, model M8 provided a significantly better fit to the data than either model M7 or model M8a (p < 0.00001 for all taxa for both comparisons), thus revealing the presence of positively selected codons along the S-domain of *SRK*. In *A. halleri*, after excluding sites with a gap in any of the sequences, the sequences spanned over 168 codons, including 33 codons from HV2, 11 from HV3 and 5 from CVR. Overall, 12 codons showed evidence for positive selection in *A. halleri *(>0.95 posterior BEB probability of belonging to the *ω *> 1 category; Fig. 3). Of these, 8 were within HV2 (273S, 274D, 276Y, 286S, 287I, 294S, 299I, 306V), none in HV3 and one in CVR (415E), the remaining 3 codons (353S, 359R, 388S) being outside HV regions. Given the relative proportion of HV regions along the sequences, this represents a highly significant clustering of sites within HV regions and CVR (p = 0.0009; hypergeometric distribution). In *A. lyrata*, 177 codon sites contained no gap in any of the sequences, and 13 sites had a high (>0.95) probability of belonging to the *ω *> 1 category (Fig. 3). Again, there was a significant clustering of these sites into HV regions and CVR (p = 0.0198). In Brassica, we reanalysed the data in [26] but restricted the dataset to the codon positions sequenced in *A. halleri *and excluded two *A. lyrata *sequences used in this study. After alignment, 195 codon sites contained no gap in any of the sequences, and 12 sites had a high (>0.95) probability of belonging to the *ω *> 1 category (Fig. 3). All of these sites except 413E were included in the [26] results, and only three of the 14 sites identified by [26] remained undetected by the present analysis (291L, 303V, 306V). Thus, although based on a more restricted stretch of sequence in the S-domain (195 *vs*. 423 codons), our results were very consistent with those of [26]. Interestingly, one of the discrepant sites (306V) was identified in *A. halleri *and *A. lyrata*, suggesting that part of the differences observed may have been due to the inclusion of the two divergent *A. lyrata *sequences in the [26] analysis. Again, there was in Brassica a significant clustering of these sites into HV regions and CVR (p = 0.0002). Strikingly, however, a large proportion of these sites in Brassica (5/12) were within HV3, while HV3 contained a single positively selected site in *A. lyrata *and none in *A. halleri*. Overall, 25 positively selected sites were detected in at least one of the three taxa (238L, 259R, 273S, 274D, 276Y, 286S, 287I, 288L, 294S, 299I, 305K, 306V, 320L, 330W, 332M, 339E, 340A, 341A, 353S, 359R, 388S, 405T, 413E, 415E and 422D; see Fig. 3). The "fixed sites" model implemented in HYPHY further confirmed that selection was stronger in HV regions and CVR than in the rest of the protein (higher *ω*), since allowing *ω *to vary between HV regions and the rest of the protein significantly improved the likelihood of the model (p < 0.001 in all three species). This difference was not due to synonymous rate variation, since allowing *dS *alone to vary between HV regions and the rest of the protein did not significantly improve the likelihood of the model (LRT with one d.f., *p *= 0.416, *p *= 0.597 and *p *= 0.103 for Brassica, *A. halleri *and *A. lyrata*, respectively).

Analysis of the subset of longer sequences in *A. halleri *including both the S- and the kinase domains using model M8 revealed that eight of the 323 codons analyzed (273S, 274D, 276Y, 287I, 299I, 345I, 415E, 421Y) belonged to the maximum likelihood "positively selected" category. Consistent with [26], all of these codons were from the S-domain, and none of them were from the kinase domain. Since only six kinase domain sequences were available in *A. halleri*, the analysis had low power and none of these sites had a posterior BEB probability of belonging to the *ω *> 1 category above 0.95.

These results were robust to both model specifications (number of categories allowed for non-positively selected sites) and uncertainties in phylogenetic reconstruction. One, 7, and 8 sites were retrieved by M8 (10 categories for non-positively selected sites) but were not retrieved by M2a (a single category) in Brassica, *A. halleri *and *A. lyrata*, respectively, while sites retrieved by M2a were in all three taxa subsets of those retrieved by M8. Thus, in all three taxa, both models returned very similar sets of sites, except that M2a returned a lower number of sites, presumably due to lower power. Results were also robust to uncertainties in the phylogenetic reconstruction. The 95% credible set of trees was of different size in the three taxa, with 5, 93 and 903 trees in *A. halleri*, Brassica and *A. lyrata*, respectively. In *A. halleri*, the maximum likelihood "positively selected" category was very similar across the five credible topologies, with only minor differences apparent in the posterior BEB probabilities (one topology identified 13 sites and two identified 14 sites versus 12 sites in the ML-tree). In Brassica, the analysis of ten trees randomly chosen from the 93 credible set consistently recovered 11 of the twelve sites identified by the BEB procedure using the ML-tree. The higher uncertainty in the phylogenetic reconstruction in *A. lyrata *(903 trees in the 95% credible set of trees) apparently led to a higher level of uncertainty in the identification of sites, since only eight of the 13 sites were consistently identified using ten randomly chosen trees from the credible set.

As shown on Fig. 3, a single site (274D) was consistently identified as evolving under positive selection in all three species, while most sites were only identified in a single species or in two different species. Of the 12 sites identified in *A. halleri*, only 2 (16.7%) were also identified in Brassica. Similarly, of the 13 sites identified in *A. lyrata*, only 3 (23.1%) were also identified in Brassica. Although the concordance between *A. halleri *and *A. lyrata *was higher (64.1%), this may still be considered very low given the close phylogenetic proximity of these two species. Interestingly, posterior *ω *estimates for these sites also differed greatly among taxa. Codon sites in Brassica with a high (>0.95) probability of belonging to the *ω *> 1 category had a BEB posterior *ω *of 2.985 (average across the 12 sites). In sharp contrast, the BEB posterior *ω*s were 1.444 and 1.486 in *A. halleri *and *A. lyrata*, respectively, i.e. about two times smaller (2.038 times smaller on average).

### Simulations

Simulations in EVOLVER revealed that this low level of concordance was indeed expected given the method's low power and the large divergence among *SRK *sequences. All sequences from the three species (65 sequences in total) were used to obtain the phylogeny shown on Fig. 1B. The maximum likelihood analysis with CODEML identified that 11.8% of the 161 sites (i.e. 19 codons) were evolving under *ω *= 1.754 >1. These values, along with the estimated codon equilibrium frequency, *ω *distribution (*p *= 0.530, *q *= 0.758 for the *β *distribution), tree length (23.47 substitutions per codon site) and transition/transversion ratio (*κ *= 1.9375), were used to simulate 100 replicates of the evolution of 65 sequences. These simulations first confirmed that the method was highly accurate at this high level of divergence. Analysing simulations run with no site in the *ω *> 1 category (model M7), we found that accuracy was indeed consistently high (0.97, 0.93 and 0.95 in Brassica, *A. halleri *and *A. lyrata*, respectively), suggesting that sites classified with high probability (>0.95) into the *ω *> 1 category were unlikely to be false positives. However, the power of the analysis was apparently low when analyzing a single taxon at a time, since only 14.8%, 34.2 and 38.5% of sites simulated as positively selected were effectively detected in Brassica, *A. halleri*, and *A. lyrata *respectively. In contrast, the multi-species screen had substantially higher power. On average, collectively considering all sites detected by at least one of the three taxa allowed us to effectively identify 59.2% of all positively selected sites. This increased power of the multi-species screen was accompanied by a slight decrease of accuracy to 0.86, still suggesting that the identification of sites in the *ω *> 1 category was reliable.

**Figure 4:**
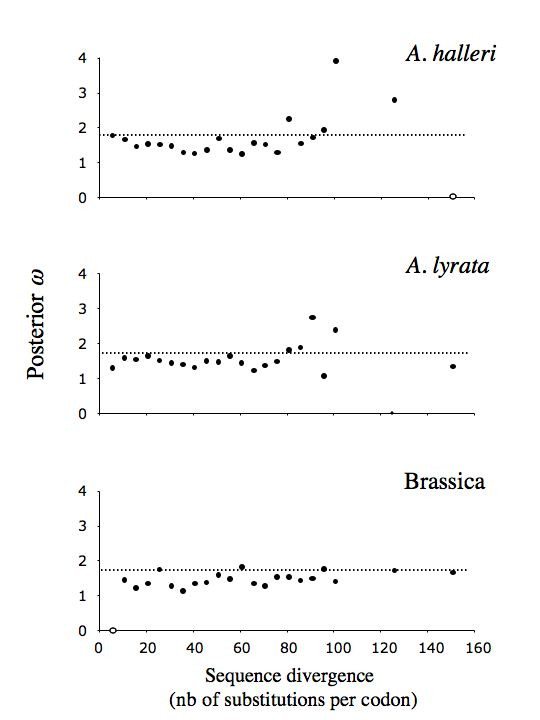
**Effect of sequence divergence on posterior *ω***. Posterior *ω *values were computed for cases where at least one site had a high (>0.95) probability of belonging to the *ω *>1 category. Results are averages over 5 independent replicates. Open circles represent situations where no site was identified in any of the five replicates. Despite a slight underestimation of posterior *ω *values (parametric value = 1.754; horizontal dashed line), there is no evidence that saturation of divergence alone could have caused the lower *ω *found in *A. halleri *and *A. lyrata *than in Brassica.

Although selective constraints were identical across the three taxa in the simulations (*i.e*. the same codons were simulated as evolving under positive selection), the match between sites identified in the three taxa was surprisingly poor and comparable to the observed match. Thus, in the simulations, an average of 47.0% of sites detected in *A. halleri *were also identified in *A. lyrata*, 16.1% of sites detected in *A. halleri *were also identified in Brassica and 17.1% of sites detected in *A. lyrata *were also identified in Brassica. None of these simulated levels of concordance differed significantly from the observed levels (*p *= 0.77, 0.57 and 0.67, respectively). Thus, separately analyzing such divergent sequences from three taxa that evolved under identical evolutionary constraints indeed led to the identification of different codon sites in the different taxa, and the observed low concordance was within the expected range. As shown in Fig. 4, there was also no evidence that sequence divergence alone could be responsible for the observed variation in posterior *ω *among the three taxa. In the simulations, ratios among estimated posterior *ω*s in the three taxa never even approached the observed value of 2.038 (*p *< 0.01).

**Figure 5:**
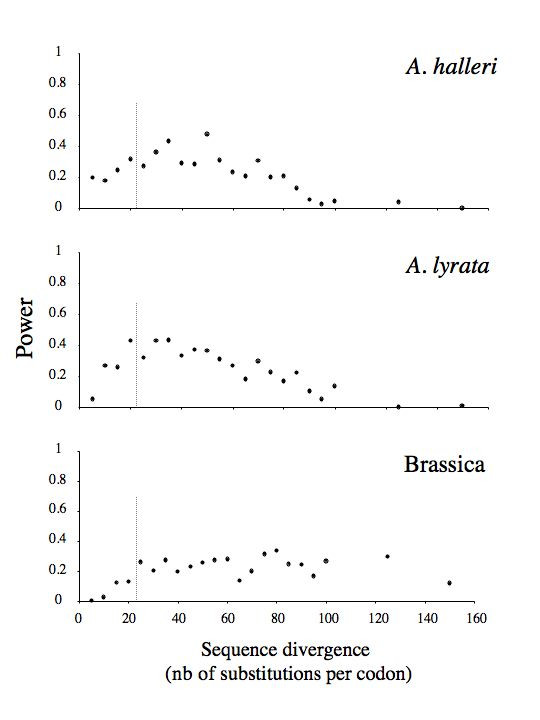
**Effect of sequence divergence on power to detect sites evolving under positive selection**. In the simulations, an average of 19 sites were assigned to evolve under positive selection (see text for simulation parameters). The level of sequence divergence estimated from the combined *A. halleri*, *A. lyrata *and Brassica sequences is 23.47 substitutions per codon. Results are averages over 5 independent replicates. The dashed vertical line represents the observed level of divergence estimated from the analysis of the 3 taxa combined.

Simulations of sequence evolution with different tree lengths confirmed that the low level of concordance was partly due to overall low statistical power. The number of detected sites remained in all cases much lower (maximum = 9 sites in Brassica and *A. halleri*, 11 sites in *A. lyrata*) than the actual number of sites simulated as evolving under positive selection (on expectation, the same 19 sites for all species), thus confirming that the method had low power (Fig. 5). Consistent with [27], our simulations showed that power was initially low when sequences were only slightly divergent (zero sites detected with sequence divergence <5 substitutions per codon), then increased at intermediate levels of divergence and then started to decrease again when sequence divergence reached saturation. Interestingly, the observed level of sequence divergence in *A. halleri *and *A. lyrata *was almost coincident with the peak of maximal power, while for Brassica, maximum power was attained at higher sequence divergence than actually observed. Tree length also had no detectable effect on accuracy, since there was no increase in the rate of false positives with sequence divergence (data not shown). These results suggested that high sequence divergence would result in lower power, but without increasing the rate of false positives.

## Discussion

### Twenty-two *SRK *sequences in *A. halleri*

Combining results from this study with those from [22], a total of twenty-two S-alleles have been identified and sequenced in *A. halleri*, together with several other members of the S-gene family previously identified in *A. lyrata*. Co-segregation and linkage to the incompatibility phenotype has been demonstrated for the eight S-alleles tested (*AhSRK01*, *AhSRK04*, *AhSRK12 *and *AhSRK21 *in [22]; *AhSRK15*, *AhSRK21*, *AlSRK14 *and *AlSRK21 *in this study). By tracing recombinants in a backcross involving a F1 hybrid between *A. halleri *and *A. lyrata *with genotype *AhSRK01*/*AlSRK21*, [31] also demonstrated that the functional S-locus is localized at a position analogous to the *ψSRK *position in *A. thaliana *[32]. These observations are particularly important because [28] showed that *SRK *sequences in *A. lyrata *do not show 100% monophyly, as two unlinked sequences (*Aly13-2 *and *Aly13-7*) were found to be nested within the *SRK *cluster. Our results confirm that these two sequences seem to be exceptions, since all other putative *SRK *sequences that have been identified and tested have proven to be linked to self-incompatibility (see *e.g*. [33]). Consistent with these sequences belonging to *SRK*, and thus evolving under strong balancing selection causing long coalescence times [34], we found that 12 of the 22 putative S-alleles in *A. halleri *form trans-specific pairs with high similarity with S-alleles sequences from *A. lyrata*. Overall, the level of diversity among putative S-alleles in *A. halleri *is comparable to that reported for *SRK *haplotypes in *A. lyrata *[35].

Indeed, this level of diversity is the highest ever-reported in *A. halleri*, both in terms of the number of different haplotypes (20 sequences here plus 2 sequences previously reported in [22] = 22 different sequences in a total of 36 individuals) and in terms of sequence polymorphism. The most exhaustive study of sequence polymorphism to date in *A. halleri *reported a mean silent nucleotide diversity *π*_*S *_= 0.015 (range [0.0025–0.0569], [36]) across eight independent genes, while we observed *π*_*S *_= 0.819 in exon 1 of *SRK, i.e*. a 54 fold increase in diversity. Analyzing the pattern of variation across the whole gene, we see no clear relationship between synonymous nucleotide diversity and distance from the S-domain, as noted already by [34] in *A. lyrata*. As we also showed that positively selected sites are only occurring in exon 1, this suggests that the high diversity observed in exons 2 to 6 is due to genetic hitchhiking and also that intragenic recombination is very low.

### How many sites evolve under positive selection?

The PAML approach has been extremely successful in revealing protein evolution in a variety of situations. Our study empirically demonstrates the limits but still usefulness of this approach in a case of extreme sequence divergence. Although the power of the analysis was low in any of the three taxa, the accuracy remained very high. This observation is consistent with results of a simulation study by [27], who revealed that the accuracy remained high and that increasing the number of lineages was the best way to increase statistical power, even at high levels of divergence. Our approach, combining sets of sites revealed by at least one taxon (25 codons revealed) was apparently more powerful than defining the set of sites from a single dataset containing all three taxa (19 codons revealed), so we conclude that 25 codon sites over 180 (13.9%) are identified as evolving under positive selection. Because of limited statistical power of the analysis, however, this represents only a fraction of all sites truly evolving under positive selection. Our simulations allowed us to estimate that the power of this multi-species screen was 59.8%. By extrapolation, we thus estimate that 25/0.598 = 41.8 of the 180 codon sites have evolved under positive selection, *i.e*. almost a quarter of the sites in the sequence could be involved in recognition. This proportion is higher than that reported in [26] in Brassica (6.38% of sites) and also higher than those recently reported in the gametophytic self-incompatibility pollen *SFB *gene in *Prunus spinosa *(23 out of 315 sites = 7.3%, [37]) and the gametophytic self-incompatibility pistil S-RNase gene in *Lycium parishii *(7 out of 130 sites = 5.4%, [38]). As we showed here, a possible explanation for this difference is that the screens used in these studies may also have had low power. Collectively, these results thus indicate that a large proportion of sites in self-incompatibility genes are probably involved in the molecular interaction determining compatibility.

### Location of sites under positive selection

Because of the low level of false positives revealed by our simulations, sites belonging to the *ω *> 1 category should be considered as reliable candidates for further functional analyses. These codons were strongly clustered in hypervariable regions, and we checked that this observation was not an artifact due to variation in evolution rate at synonymous sites. Our results thus support previous claims of the functional importance of hypervariable regions [14-21,25]. Interestingly, however, their distribution across HV2, HV3 and CVR differed strongly among the three taxa, with HV3 containing most sites detected in Brassica, while this region contained a very low number of positively selected sites in *A. halleri *and *A. lyrata*. In line with [9], this raises the intriguing possibility that functionally important parts of the proteins may differ along different allelic lineages. However, although our simulation approach demonstrated that the number of false positives remained very low, it also demonstrated that such a poor concordance among sites identified in different taxa is consistent with the analysis of highly divergent sequences.

### More intense selection in Brassica ?

Our study also revealed that the observed higher posterior *ω *estimate in Brassica as compared to *A. halleri *and *A. lyrata *was outside the expected range of random variation computed from simulations, possibly suggesting more intense selection in Brassica. How should higher posterior *ω *in Brassica as compared to *A. halleri *and *A. lyrata *be interpreted biologically? All three taxa most probably inherited a common set of alleles from their ancestor. Although the number of alleles is comparably high in the three taxa, Brassica alleles cluster in two main lineages, whereas all *A. halleri *and *A. lyrata *alleles are highly divergent from one another. A possible explanation for this difference is that the genus Brassica went through a severe bottleneck that drastically reduced the number of alleles. As noted by [39], the intensity of balancing selection on an S-locus is inversely related to the number of alleles in a population. Thus, balancing selection may have been very intense, leading to strong selection for allelic diversification shortly after the putative bottleneck. How strong a bottleneck can generate such a difference in posterior *ω *remains to be determined.

### Balancing *vs*. positive selection: exploring the limits of PAML

Although phylogenetic methods to detect natural selection on protein coding sequences have initially been developed for between-species comparisons, there is an increasing interest in adapting these methods to the analysis of intraspecific polymorphism. Our data can be used to evaluate some limits of this approach.

A first limit is that our simulation procedure assumed a constant *ω *for each codon across the whole phylogeny. This is unlikely to be true, since frequency-dependent selection implies that an allele's selection regime varies together with its frequency. Indeed, a newly arisen allele initially experiences positive selection as long as it remains below its equilibrium frequency. Once equilibrium has been reached, the allele is then actively maintained by balancing selection, not positive selection anymore. *ω *values are thus expected to change along each branch of the phylogeny, such that their precise interpretation is unclear in the context of frequency-dependent selection.

A second limit is that our approach assumes no recombination. Simulations by [40] have shown that the presence of recombination affects the power to detect selection because PAML assumes a single tree for the whole protein, whereas recombination leads to different gene genealogies in different regions of the gene. Currently available methods to estimate jointly recombination and selective pressures along the gene (e.g. [41]) rely on explicit population genetics models, while we obtained a single sequence for each *SRK *haplotype in the whole species. Accurate allele frequency data are thus not available, currently preventing us from taking recombination formally into account. Although we acknowledge that this will be important once appropriate data are available, we think that recombination is probably not causing bias in the analyses for the following reasons. As shown by [40], PAML is indeed relatively tolerant for a certain range in the recombination rate, especially with models M7 and M8. In addition, the existence of separate genes for the pollen and pistil recognition functions apparently causes suppressed recombination to maintain coadapted sets of the two different loci. Accordingly, comparison between physical and genetic maps of the chromosome carrying the S-locus indeed showed reduced recombination in the SI region in *A. lyrata *[42]. In line with [35], reduced recombination is compatible with our observation of no significant decrease of LD across the S-domain as well as with a strong hitchhiking effect on synonymous nucleotide variation throughout the kinase domain of *SRK*.

A third limit of the parametric bootstrap approach used to investigate power and accuracy is that it relies on parameters estimated from the data. Given the important divergence observed among sequences, saturation is likely to occur and divergence is probably the most poorly estimated parameter of our analysis. In particular, the length of the long internal branches of the tree used to simulate sequence evolution is probably poorly estimated. In addition, the three taxa analyzed have very contrasted allelic phylogenies. Brassica has a single long internal branch separating the two dominance classes, while *A. lyrata *and *A. halleri *have numerous long branches, so the number of non-synonymous substitutions may be underestimated more severely in *A. lyrata *and *A. halleri *than in Brassica, thus providing a potential explanation for the lower *ω *in *A. halleri *or *A. lyrata *than in Brassica. Could uncertainty in branch length estimation have affected our conclusions? Our simulations revealed that power declined with sequence divergence more quickly than accuracy of the posterior *ω *estimation. Thus, the signal of positive selection was lost before any bias in the BEB posterior *ω *estimation could be detected. Our simulation approach mimics the generation of real datasets, such that any bias due to saturation would be present in both real and simulated datasets, making the comparison robust to such effects.

## Conclusion

This is the first report of sequence polymorphism at the female specificity determinant gene *SRK *in the self-incompatible *Arabidopsis halleri*. Sequence polymorphism was extremely high, and twenty-five codons showed signs of positive selection in either *A. halleri*, *A. lyrata *or Brassica. Using simulations, we demonstrate that the signature of balancing selection can be identified reliably at the codon level and estimate that over 20% of all codons in the S-domain may actually be involved in recognition. Brassica had higher posterior rates of non-synonymous to synonymous substitutions than either *A. halleri *or *A. lyrata*, possibly suggesting that allelic diversification in this genus was both more recent and more intense than in either *A. halleri *or *A. lyrata*.

## Methods

### Plant material

A total of 36 *A. halleri *individuals was screened for *SRK *polymorphism. Individuals were collected from 17 widely distributed European populations (Table 1). A single individual was analyzed per population, except in Auby, where 16 individuals were analyzed and in AL08 and TC05, where two individuals were analyzed (Table 1). Fresh leaves were dried overnight at 55°C and DNA was extracted using the extraction kit Dneasy^® ^from Qiagen^®^.

### Molecular methods

In line with the notation proposed by [33], *AhSRKx*, *AlSRKx*, *BoSRKx *and *BrSRKx *refer to haplotype *x *in *A. halleri*, *A. lyrata*, *B. oleraceae *and *B. rapa*, respectively. Nearly full-length sequences for *AhSRK04 *and *AhSRK10 *were taken from [22]. *A. lyrata *sequences (n = 24) were taken from [33] and Brassica sequences (n = 19) from [26]. *SRK *polymorphism in *A. halleri *was first screened using the S-domain only.

Because of the lack of fully conserved regions along the S-domain, several primer pairs were used in an attempt to identify the largest possible set of different alleles (primer sequences and amplification conditions in [18]). 13SEQ2F and SLGR were used as forward and reverse primers, respectively, for all individuals, and 13SEQ1F and SLGF were used as forward primers in combination with SLGR for a subset of individuals where either a single or no allele was identified with 13SEQ2F-SLGR. Amplification products were ligated into plasmids and transformed into chemically competent bacteria using the TOPO TA cloning kit (Invitrogen Corporation) according to the manufacturer's protocol. An average of 8 positive clones per individual (1–45) were sequenced using the vector's M13 forward and reverse sequencing primers and BigDye3.1 (Appplied Biosystems) sequencing kit on an ABI3100 capillary sequencer. Sequences were edited using MEGA3 [43] and searched with BLASTN against a local library of sequences containing all published sequences of the *S*-family in *A. lyrata *[22,33,34,44]. Sequences whose closest match was one of the *A. lyrata *alleles demonstrated to be linked to the SI phenotype were provisionally considered to also be *SRK *alleles in *A. halleri*. Among those, sequences with a maximum divergence of 8.1% (average *A. halleri*-*A. lyrata *divergence across 10 unlinked genes; [22]) were considered as "matching alleles" of either ancestral origin or introgression. Sequences whose closest match was a member of the gene family in *A. lyrata *were considered as paralogues. Since *Aly13-2 *and *Aly13-7 *in *A. lyrata *were found to be independent from the SI phenotype [18], matches to these sequences were excluded. To correct for PCR errors, positive clones were then screened further until at least three replicates were obtained for each of these sequences. Majority-rule consensus sequences were obtained by manual correction.

Evidence for the presence of a kinase domain linked to the S-domain was then searched in several of the putative S-alleles using allele-specific forward primers designed from the S-domain sequence in conjunction with general degenerate reverse primers from the kinase domain : *R4997 *: CACCKYGARTCTTKGTGAAG or *R4694 *: AVATTTTCCAAGTAYTCRTA designed from an alignment of the published *SRK *kinase domain sequences in *A. lyrata*. The PCR products obtained were sequenced directly as reported above, using intermediate internal sequencing primers to tile the sequences entirely.

### Segregation analyses

We tested for co-segregation of two pairs of putative *SRK *haplotypes among offspring obtained from an interspecific cross between *A. halleri *(pollen donor) and *A. lyrata *(pollen receiver). Seeds were allowed to germinate in small pots and seedlings were harvested, dried and DNA was extracted as described above. Offspring were genotyped using primers specific to each of the four *SRK *haplotypes of the parents (V. Castric, unpublished results). To test for an association between putative *SRK *genotypes and incompatibility phenotypes, we performed controlled pollinations among offspring of this cross and estimated compatibility of the genotypes through measurements of fruit-set.

### Structural and diversity analysis

Nucleotide sequences were trimmed to the shortest and translated into amino acid sequences in MEGA3.1. To preserve the amino acid numbering of the sequence, *A. halleri *and *A. lyrata *amino acid sequences were all aligned to the alignment profile used in [26], kindly provided by R. Sainudiin) using CLUSTALX [45] followed by manual adjustments in the alignment editor in MEGA3.1. In accordance with [26], codon positions in the alignment were numbered from the ATG codon of *BoSRK60*. N-linked glycosylation sites along the amino acid sequences [46,47] were identified using N-GLYCOSITE [48,49]. Variation of amino acid diversity was investigated along the sequence by sliding a 5 amino acids window with a step of 1 amino acid. We tested for recombination by comparing the relationship between a measure of linkage disequilibrium (r^2^) and the distance between polymorphic sites, as implemented in the software package LDHAT 2.0 [50]. Significance of the correlation was determined from 1,000 permutations of the variable sites.

### Sites under positive selection

Sites evolving under positive selection were searched for using the codon-based model implemented in CODEML in the PAML analysis package [51] using the "complete deletion" option, *i.e*. only sites with no indel in any of the sequences were included in the analysis. Briefly, a maximum-likelihood phylogenetic tree was constructed using DNA-ML from the PHYLIP package [52] for sequences of each taxon separately, as well as for combined datasets including all three taxa. Given this topology, the log-likelihood of models M7, M8a and M8 of [53] were compared using a likelihood ratio test (LRT). Models M7 and M8a allow the different codon sites along the sequence to evolve under a range of beta-distributed *ω *ratios (0 <*ω *< 1 for M7 and 0 <*ω *≤ 1 for M8a), thus simulating a range of constraints by purifying -but not positive- selection. M8 includes all these features, but has an additional category for sites with *ω *> 1, thus allowing for positively selected sites. Given the proposed tree topology, CODEML uses Felsenstein's pruning algorithm to sum over all possible ancestral states and maximize the overall likelihood of model M8 for the observed sequences. This procedure thus provides maximum likelihood estimates for the codon substitution model, the transition/transversion ratio *κ*, the overall number of nucleotide substitutions per codon (tree length) and the distribution of replacement *vs*. silent substitutions *d*_N_/*d*_S _= *ω*. Since model M7 is nested within model M8, a LRT with two degrees of freedom was used to determine whether M8 provides a comparatively better fit than M8, and thus whether positively selected sites occur along the sequence. A LRT with one degree of freedom was used to compare M8a with M8 [54]. The Bayes Empirical Bayes (BEB) procedure [55] was then used to estimate at each site the probability that it belongs to the *ω *> 1 category. Sites having a probability >0.95 of belonging to this category were referred to as "positively selected" sites. We compared the strength of selection between hypervariable regions and the rest of the protein using two approaches. We first used the hypergeometric distribution to determine whether these sites clustered within hypervariable regions. We then used HYPHY V.0.99 [56] to compare the likelihood of a model allowing different *ω *for HV regions and the rest of the protein with that of a model constraining them to be equal (the "fixed-sites" approach described in [57], LRT with one degree of freedom). We also used HYPHY to test for differences in rate of synonymous substitutions between HV regions and the rest of the protein, as such differences could lead to misidentification of sites as positively selected [58]. The locations of positively selected sites in *A. halleri *and *A. lyrata *were compared with those obtained from the analysis in Brassica. The level of pairwise concordance was determined as the average proportion of sites retrieved in a taxon that were also retrieved in the other taxon. For each taxon, posterior *ω *were averaged over all positively selected sites. As advocated in [27], robustness to the exact model specification was assessed by comparing sites retrieved by M8 with those retrieved by M2a, a simpler model where all sites evolving under purifying selection belong to a single category. The sensitivity of the method to the exact tree topology was assessed by running M8 under a subset of trees of the 95% credible set of trees obtained in MRBAYES version 3.1 [59], implementing a general reversible time 4×4 nucleotides transition model with equal rates across sites. Two independent MCMC chains were run for 1,000,000 iterations and trees were sampled every 100 iterations after discarding the first 2,500 for burn-in.

### Simulations

The following parametric bootstrap approach was then used to determine whether the observed concordance between sites retrieved by all three taxa was indeed expected given the observed phylogeny. The program EVOLVER in the PAML package was used to simulate 100 replicates of sequence evolution with a fixed proportion of sites evolving under positive selection. All parameters used for these simulations, including the number of codons evolving under positive selection, were assigned their maximum likelihood estimates under model M8 obtained using the whole dataset that included all three taxa (22, 24 and 19 sequences in *A. halleri*, *A. lyrata *and Brassica, respectively, *i.e*. 65 sequences total). The simulated sequences obtained were then separated into three subgroups corresponding to the three taxa and were analyzed separately with the same CODEML procedure as described above. Given that sites evolving under positive selection in the simulations were identical across all three taxa, we expected that the analysis in each taxon should consistently retrieve the same sites. Thus, the level of pairwise concordance of each simulated dataset was compared to that of the real data. In addition, since sites evolving under positive selection were known in the simulations, these simulations enabled us to quantify the statistical power of the BEB procedure when model M8 was analyzed with sequences from a single taxon. Following [27], power was defined as the proportion of sites actually evolving under positive selection that the analysis was able to detect. Accuracy was defined as 1 minus the rate of false positives and assessed by analyzing with model M8 100 replicate simulations where no site evolved under positive selection (model M7). Power and accuracy were also investigated using all sites detected by any of the taxa, *i.e*. on the combined set of sites across all three taxa independently. Because the results revealed large differences among taxa in posterior BEB *ω *estimations (averaged across sites classified in the *ω *> 1 category), the simulated sequences used to assess power were also used to determine whether such differences were compatible with a null model where all sequences evolved according to the same *ω *distribution.

We then investigated the effect of decreasing or increasing sequence divergence on power, accuracy and the posterior *ω *estimate. We simulated different levels of sequence divergence by varying the overall number of nucleotide substitutions per codon (tree length 5–150 nucleotide substitutions per codon), thus simulating evolution for shorter or longer periods of time. Simulations were run along the fixed ML-topology including all three taxa, and datasets were analyzed separately for each taxon. Five and 150 nucleotide substitutions per codon site distributed over the whole 65-sequences phylogeny were chosen to represent realistically low and high extremes of sequence divergence, respectively. Power, accuracy and posterior *ω *estimate results were determined and averaged over five independent replicates for each level of divergence.

## Authors' contributions

XV and VC conceived the experiment. VC carried out the molecular genetic studies and performed the statistical analysis. VC and XV drafted the manuscript.

## Supplementary Material

Additional file 1 Sequence alignment. *AhSRKx*, *AlSRKx*, *BoSRKx *and *BrSRKx *refer to haplotype x in *A. halleri*, *A. lyrata*, *B. oleraceae *and *B. rapa*, respectively. Codon positions in the text are given in comparison with the full *BoSRK60 *(start codon = 1). Hypervariable regions HVR1, HVR2, HVR3, and CVR established by Nishio and Kusaba (2000) correspond to residues 205–220, 270–306, 328–343, and 413–423, respectively. Click here for file
